# Protocol of a randomized controlled trial of hearing protection interventions for farm operators

**DOI:** 10.1186/s12889-015-1743-0

**Published:** 2015-04-18

**Authors:** Marjorie C McCullagh, David L Ronis

**Affiliations:** University of Michigan School of Nursing, Ann Arbor, MI USA

**Keywords:** Hearing loss prevention, Hearing conservation, Farmers, Randomized controlled trial

## Abstract

**Background:**

Hearing loss and tinnitus are prevalent in America, and noise-induced hearing loss is a leading cause of hearing loss. Noise-induced hearing loss has negative impact on quality of life, physical and emotional functioning, social life, and employment. In addition, noise-induced hearing loss results in heavy social and economic burdens on families and communities from all ethnic and socioeconomic groups. Farmers are a group that is particularly high risk for noise-induced hearing loss, and is underserved by programs designed to limit that risk. They are among the most noise-exposed group of workers, and experience the second highest prevalence of noise-induced hearing loss among all occupational categories. In agriculture, 1.5 million workers (43.3%) report exposure to hazardous noise. Although use of hearing protection devices (HPDs) would protect them from noise-induced hearing loss, use among farmers is low.

**Methods/Design:**

The purpose of this project is to compare the effectiveness of several approaches to influencing hearing protector use. Approaches include: a) an interactive, predictors-based intervention delivered via the Internet; b) a static informational web site; and c) a mailed sampler of hearing protectors. The goals are to further develop an intervention to promote farmers’ use of HPDs, and compare the effectiveness of the interventions delivered in various combinations. Participants will include 701 farmers. Sites will be affiliates of a major farmer organization. Data will be collected at baseline, 6, and 12 months. A random intercept mixed model will be used to explore the fixed effects of the three NIHL prevention interventions over time while adjusting for age and gender. This project will involve a partnership between the University of Michigan and a major farmer organization to accomplish project aims.

**Discussion:**

Results of this study will be used to inform future research-to-practice studies to increase hearing protector use. Increased use of hearing protectors is expected to reduce rates of noise-induced hearing loss and other negative effects of high noise exposure, and improve quality of life in this high-risk and underserved group.

**Trial registration:**

Clinicaltrials.gov NCT01454895 Registered 14 October, 2011.

## Background

Noise-induced hearing loss (NIHL) is highly prevalent among US workers, particularly among farmers. An estimated 22 million workers are exposed to hazardous noise at work [1] and NIHL is among the most common work-related diseases, and the second-most self-reported occupational disease or injury [2]. Estimates of prevalence rates for NIHL among farmers vary greatly, and have been reported to be 17% [3], 22% [4], 38% [5], 65% [6], and 72% [7]. In comparison studies, farmers were more likely to have hearing loss than non-farmers [3,6,8].

NIHL is characterized by loss of hearing in higher frequencies; it is permanent and incurable, and typically progresses slowly and insidiously with continued exposure to high levels of noise. Most people are unaware that they are affected until it is already moderately severe [9].

NIHL has negative impact on the quality of life of the affected individuals as well as their families and communities, affecting physical and emotional functioning, social life, and employment. In addition, persons with NIHL frequently experience tinnitus, and have increased safety risks due to difficulty hearing warning sounds [10]. Importantly, hearing loss has also been associated with increased risk for injury among farmers [11].

Treatments for NIHL are limited and unsatisfactory, indicating that primary prevention offers the best option for success. Because NIHL is permanent and irreversible, treatment is limited to hearing aids for sound amplification. Most users find hearing aids expensive, unlike their natural hearing, and particularly unsatisfactory when there is background noise or when trying to focus on one speaker when there are other competing sounds [12].

Systems to protect workers from NIHL are not present in the farm work setting. Farmers are unique in that unlike workers in general industry, most farmers are not protected by the OSHA Hearing Conservation Standard (i.e., noise level monitoring and a hearing conservation program for at-risk employees which includes audiometric testing, training, and provision of hearing protection devices) [13]. Also, because most farms in the US are small, family-run organizations, there is no labor advocacy for worker hearing health and work-based health programs [14]. Many farmers may underestimate their exposure to noise hazards and consequences of noise exposure, and may not be knowledgeable about NIHL prevention techniques.

Noise elimination is the most preferred method of prevention of NIHL. However, this approach is often not technically or economically feasible in the farm work environment [14]. Consistent use of hearing protection devices is effective in preventing NIHL [15-17]. While there are several types of hearing protectors marketed (e.g., foam plugs, ear muffs), there is no “best” type of hearing protection; the “best” is the one the user prefers and will wear.

The problem of NIHL has been identified as a priority by federal agencies, including Healthy People 2020 [18], the National Institute for Occupational Safety and Health [19], and the National Institutes for Deafness and Communication Disorders [20].

There is evidence that interventions are needed, and can be successful in relieving this problem without negative impacts and at low costs. Use of hearing protectors is an effective method of preventing NIHL when engineering or administrative controls are not feasible or economical [21-23]. Epidemiological studies show that the need for interventions to increase hearing protector use among farmers is high, and that unlike some other worker groups, there is no ceiling effect limiting effectiveness of interventions [24-26]. Studies have identified predictors of hearing protector use among farmers, and have demonstrated that farmers are interested in increasing their use of hearing protectors [25]. Predictors-based and Internet-based interventions have been effective in other groups [27-31], and the proposed study seeks to apply this existing research to a test of interventions for farmers, a high risk and underserved group.

The proposed study takes advantage of existing organizations of farmers by partnering with the single largest farm organization in the US. This partnership will serve to optimize recruitment and retention of participants while minimizing costs. Members of the farm organization are farm operators; many are employers of farm laborers and therefore influential in determining the use of hearing protectors by laborers.

The proposed study will answer important questions in development of behavioral interventions to widely dispersed populations. It will connect with participants through their trade association together with using Internet-based health information. These methods have potential for reaching an otherwise difficult-to-reach rural and widely dispersed population. Together with an efficacious intervention, the program has potential for excellent health impact. Results from the proposed study will provide a model for future behavioral intervention research in a dispersed population and create new approaches for the prevention of NIHL, a serious preventable impairment.

### Theoretical framework: the farmers’ use of hearing protection model

**Figure 1:**
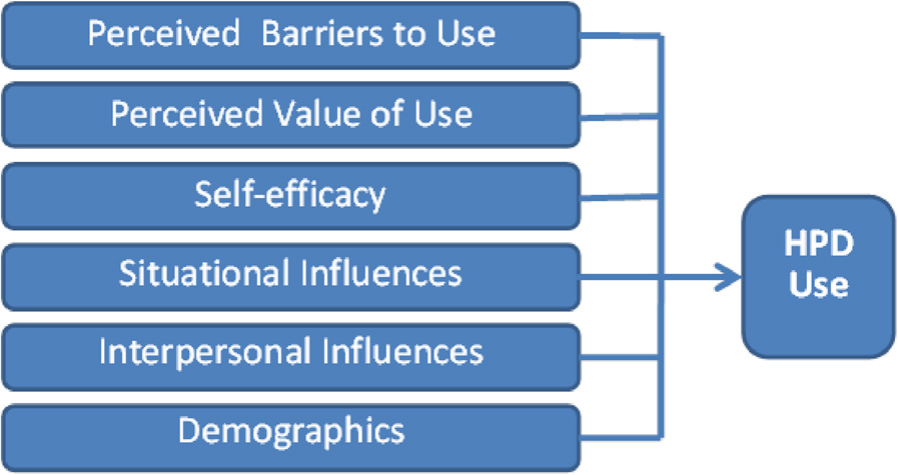
Predictors of farmers’ use of hearing protection model.

A variety of factors have been found to be predictors of HPD use in multiple studies of workers, including farmers. These factors are included in the Farmers’ Use of Hearing Protectors Model (Figure 1), and serve as a guide for the proposed study. In a previous study [25], this parsimonious model was effective in predicting HPD use in 74% of cases.

Perceived Barriers to HPD Use are impediments [32] resulting in discomfort or difficulty in communication, and have consistently shown a negative and significant relationship to HPD use in studies of factory workers [33], construction workers [32,34], and farmers ([24,25,35], McCullagh, MC.: Farmers’ preference for use of hearing protectors, unpublished), Perceived Benefits of HPD Use are users’ ideas about the positive consequences of HPD use. Benefits were found to be positively related to HPD use in studies of factory and construction workers [32,33] and farmers [24]. Availability (and accessibility) of HPDs have had a positive and significant relationship to HPD use in studies of industrial workers [33] and farmers [24,25,35]. Self-efficacy of HPD Use is the extent to which an individual has confidence in one’s own ability to use HPDs [32], has had a positive and significant relationship to HPD use in studies of factory [33] and construction workers [32]. Two studies of farmers [24,25] showed a positive, but non-significant relationship between self-efficacy and HPD use. Interpersonal Influences are the individual’s perceptions of the behaviors, beliefs or attitudes of others, operationalized in three subscales: Interpersonal Norms, Modeling, and Support [32]. Interpersonal Norms are the respondents’ beliefs about how much others think they should wear hearing protection. Interpersonal Support refers to encouragement or praise from family, friends, and coworkers about the respondents’ use of hearing protection. Interpersonal Modeling is how much respondents believe family members and other farmers use hearing protection when exposed to noise. Administration of the Farmers’ Interpersonal Influences on Use of HPDs Scales (“Norms,” “Modeling,” and “Support”) showed significant and positive relationships to HPD use in the convenience sample of farmers [24], but not in a random sample of farmers [25].

Demographic Factors (age and gender) are non-modifiable factors, but important to assess. In one survey of farmers [24], women reported use of HPDs more frequently, while other studies [25,36] indicated men are more frequent users. Age was found to have a non-significant relationship to HPD use in two studies [24,25]. There are no published studies examining the relationship between race or ethnicity and HPD use.

A limited number of studies have found other factors (e.g., noise annoyance [37], perceived susceptibility to NIHL [37], perception that health problems were preventable [35]) to have a significant relationship to HPD use in non-farming samples. Perceived negative effects of NIHL on family was significantly related to HPD use in a sample of farmers [26].

## Methods

### Overview of design and methods

The purpose of this project is to identify interventions that will increase the use of HPDs, thereby reducing NIHL. The specific aim of this study is to contrast the effects of three alternative strategies (“Interactive Web,” IWI a “Static Web,” SWI, and a sampler of HPDs, HPD I) in various combinations, on HPD use and use-related attitudes/beliefs. The study uses a randomized-controlled design. It will be conducted in partnership with a major farmer organization. The University Health and Behavior Science IRB reviewed the study protocol and determined that it is exempt from ongoing IRB review.

### Sample, enrollment, and retention

Participants in this study will be farm operators age 18 or older, who are active in production at least 20 hours per week on average, have ability to read English, and have computer and Internet access. A farm operator is the person who runs the farm, making the day-to-day decisions [38].

One or more study team members will attend selected farm trade group meetings to recruit subjects. Recruitment will continue until the enrollment quota is met. Prospective subjects will log onto a designated Web site, where the enrollment process will be managed. In addition to the data collected from direct user entry during enrollment, metadata will also be collected for use in later analysis.

### Random assignment to study conditions

After participants provide informed consent and enroll in the study, they will be assigned to the five study conditions (Table 1) by a random assignment of treatments in blocks produced by Stata software [39]. Recruitment will continue until the enrollment quota is met, resulting in five approximately equal-sized groups.

**Table 1:** **Study Conditions**

**Group**	**Observation**	**Intervention**			**Observations**
		**Interactive web information**	**Static web information**	**HPDs**	
1	Pretest	X		X	Post-tests at 6 & 12 months
2	Pretest	X			Post-tests at 6 & 12 months
3	Pretest		X	X	Post-tests at 6 & 12 months
4	Pretest		X		Post-tests at 6 & 12 months
5	Pretest			X	Post-tests at 6 & 12 months

### Follow-up

At 6 and 12 months, participants in all five groups will receive an invitation by e-mail to log on to complete post-tests online.

### Interventions

The study compares five prospective parallel groups receiving three distinct interventions, delivered in various combinations (Table 1). Participants in Groups 1 and 3 will be offered (Interactive or Static) Web-based information and, following verification of completion, a sampler of HPDs will be supplied to them. Participants in Groups 2 and 4 will be offered (Interactive or static) Web-based information and asked to verify completion. Participants in Group 5 will simply receive a sampler of HPDs on completion of the pretest.

#### Interactive Web Intervention (IWI)

This intervention will include targeted messages focusing on farmer-friendly techniques for adopting use of HPDs, interactive learning techniques (e.g., sound level meter), role modeling techniques (e.g., farmer testimonials), and cognitive, demonstration, persuasion techniques. Intervention delivery is not linear; participants will select the sequence of features they visit, as well as the time spent in each feature and number of visits to the site. Their patterns of use will be tracked by the enrollment and data collection systems and used in analysis.

The standard (“static”) information promoting HPD use delivered via Internet will consist of a previously developed informational brochures (i.e., *Have You Heard?* [40] and *They’re Your Ears Protect Them* [41].

The proposed study will provide a sampler of HPDs to a sub-sample of each group of farmers receiving the Interactive and Static Web Interventions and to a group of farmers who do not receive a web based intervention. The sampler will include the most commonly-used types of devices (i.e., muffs, foam plugs, pre-molded plugs, and semi-aurals).

The study will include development of the informational Web site, inclusive of elements previously developed (e.g., video testimonials and HPD insertion techniques, animated graphics). Near final systems will be tested with a sample of the target population (confirmative testing interviews). Fidelity of the intervention is assured because delivery of information via the Internet ensures a consistent presentation of the Interactive and Static interventions.

### Outcome measures

Outcome measures will include self-reported frequency of HPD use and related attitudes/beliefs, measured at 6 and 12 months post-intervention. The outcome variables for the study are based on the Predictors of Farmers’ Use of Hearing Protection Model and include cognitive and affective factors that are specific to the behavior of HPD use (i.e., frequency of HPD use, perceived barriers, self-efficacy, access, interpersonal influences).

The HPD Use instruments (Appendix G) measuring the concepts from the theoretical model, together with their corresponding alpha reliability coefficients are described in Table 2. Development of these scales included pretest, revision, and review for construct validity by an expert panel; the process is described elsewhere [24,36]. All have alpha coefficients near or above .70. Additional variables measured will include demographic characteristics, functional hearing ability, satisfaction with the intervention, and social desirability bias (short form of the Marlowe-Crowne social desirability scale) [42].

**Table 2:** **Scale Means, Standard Deviations, and Alpha Coefficients**

**Scale name**	**Number of items**	**Range of scores**	**Mean**	**Cronbach’s alpha**
Barriers^a^	13	1-6	2.61	.81
Benefits^b^	5	1-10	8.88	.82
Self Efficacy^a^	11	1-6	4.43	.75
Situational Influences^a^	11	1-6	4.07	.81
Norms^c^	4	1-3	2.33	.68
Modeling^d^	4	1-5	2.27	.81
Support^e^	4	1-3	1.41	.69
HPD Use^f^	4	0-100	27.50^g^	.89
Social desirability^h^	8	8-24	.^i^	.74

^a^Rating scale (1=strongly disagree; 6=strongly agree); ^b^Rating scale (1=slightly important; 10= highly important); ^c^Rating scale (1=not at all; 3=a lot); ^d^Rating scale (1=never; 3=usually); ^e^Rating scale (1=never; 3=often); ^f^HPD = hearing protection device; percent of time of use; ^g^43.2% report zero use; 56.8 percent report some use; ^h^Rating scale (1=yes; 2=no); ^I^not determined for this population.

### Analysis and sample size estimates

Power analysis was conducted using PASS software [43] to determine the sample size needed to provide 80% power to detect important effects that we expect to see in the research, based on comparisons between conditions with alpha of .05 two tailed, given findings from past studies. Given how low use of HPDs is among farmers, we expect to produce and detect changes of 13% in percent time use between conditions with a standard deviation of .3, thus an effect size d of .43 which is a bit smaller than what Cohen (1992) defined as medium sized. A sample size of 85 per group is needed to have 80% power to detect an effect size d = .43. With a second posttest at 12 months we have allowed for a loss of up to 40%, so will recruit 709 subjects total to retain 425 or 85 per group.

### Data analysis plan

Descriptive statistics will determine frequency distributions, percentage distributions, and means and standard deviations. Distributions of variables will be examined to detect and correct values outside of the legal range. They will also be examined for the normality of their distribution and transformed as appropriate so they meet the assumptions of the statistical analysis. Cronbach’s alphas will be used to assess internal consistency of multiple item scales as a measure of reliability. Scales with alphas of at least .63 will be considered usable. The various experimental groups will be compared on baseline values of variables. Independent variables found to differ between groups at baseline will be added to the analyses as covariates.

A random intercept mixed model will be used to explore the fixed effects of the three NIHL prevention interventions over time adjusting for age and gender as covariates. The model will include a random intercept for subjects to control for non-independence of repeated measures. A compound symmetric covariance structure will be specified in the model. Each of the outcomes: use of HPDS, attitudes, and beliefs will be modeled separately in order to investigate the effects of web interventions and mailed hearing protection devices. The data will be analyzed within two research designs. First will be the complete factorial design 2 (interactive vs. static web) x 2 (sent HPDs) x 3 (times: baseline, 6 months, and 12 months). This will not include the condition in which participants are simply sent HPDs. The second design will include all conditions in an incomplete factorial 3 (interactive web vs, static web vs. no web) X (sent HPDs) x 3 (times: baseline, 6 months, 12 months). SAS 9.3 (SAS Institute Inc., Cary, NC, USA) and SPSS 22 will be used for all analyses. A p-value of < .05 will indicate statistical significance.

#### Tests of hypotheses 1 and 2

1. Participants receiving interactive Web interventions will have higher HPD use and more favorable use-related attitudes/beliefs than participants not receiving Interactive Web interventions;
2. Participants receiving the mailed HPD Intervention will have higher HPD use and more positive use-related attitudes/beliefs than participants not receiving the mailed HPD Intervention.

The dependent measures used for all the aims are quantitative variables: the individual’s percentage of time using HPDs, and relevant attitudes/beliefs, as outlined in Table 1. Either before or after transformation they will be normally distributed so will be analyzed by linear random intercept mixed models.

All analyses to test Hypotheses 1 and 2 will include baseline data collection and will focus on the tests of interactions of time with web format and with sending HPDs. Tests of these interactions with time will indicate whether changes over time are equivalent in different groups, for example whether participants exposed to the interactive web intervention increased use of HPDs more than participants exposed to the static web intervention. Follow-up analyses will present mean use and mean scores on attitudes/beliefs by time and condition as estimated by the mixed model. The same analysis focusing on different interactions with time will be conducted to test hypothesis 2. Naturally, they will test the interactions of time with provision of HPDs to test the hypothesis that provision of HPDs will increase use and improve use-related attitudes/beliefs.

#### Test of hypothesis 3: participants visiting the Web site more frequently will have higher HPD use than those visiting less frequently

We will assess the number of times that each individual accesses the Website and test the effect of number of views (separately for the Interactive Web Intervention and Static Web Intervention). We suspect that people with more exposures to the Website will show more impact of it (for both the Interactive Web Intervention and Static Web Intervention). However we do not expect the number of visits to be great so do not expect to have power to detect these effects. The method of analysis will depend on the distribution of number of visits. If there is little variability in number of visits this will be treated as a dichotomous predictor variable. If more variable, it will be treated as a continuous predictor. Whether significant or not, results will provide evidence about how the Interactive and Static Web Interventions work.

#### Test of hypothesis 4. There will be no interaction between intervention delivery mode (interactive vs. Static) and the mailed HPD intervention

Analyses to test Hypothesis 4 will be conducted by a 2 (Interactive Web vs Static Web) x 2 (providing HPDs) x 3 (times: within subjects) random intercept linear mixed model. The condition of mailed HPD intervention will not be included in these analyses. As with analysis of the other hypotheses, the focus is on the interaction(s) with time. Inclusion of a pretest time in the design and analysis means that the analysis is really about changes over time. In analyses to test Hypothesis 3, the key question is whether the changes over time are different in the group that receives both the Interactive Web Intervention and HPDs than would be expected by adding the effects of Interactive Web Intervention and supply of HPDs. The significance test of the three-way interaction will test this. We hypothesize that this three-way interaction will not be significant. However a significant interaction would be interesting, too.

## Discussion

The proposed study is novel in that it focuses on a population in which there is no previously reported randomly controlled trials promoting HPD use. It is also unique in that it compares the effectiveness of previously untried (targeted, Web-based, mailed) interventions to increase HPD use.

This RCT test of interventions is designed to determine the most effective approach to increasing participants’ HPD use. The moderately large, randomly-selected sample offers an opportunity to compare effectiveness of several approaches, and to generalize results to the larger population. Results will also determine the need for future program modifications, e.g., test of booster(s). Further, given a successful test of the new interactive intervention in this project, there is potential for dissemination to reach a larger number of farmers. Results of this study will inform future Web-based interventions, interventions aimed at dispersed populations, and interventions to increase use of personal protective equipment.
